# DexGraspDiffuser: Target-Coupled Grasp and Action Diffusion for Dexterous Grasping

**DOI:** 10.3390/biomimetics11070465

**Published:** 2026-07-02

**Authors:** Juncheng Zhu, Haotian Yang, Zhile Yang, Yuanjun Guo

**Affiliations:** 1 Faculty of Data Science, City University of Macau, Taipa, Macau 999078, China; d23092100281@cityu.edu.mo (J.Z.); d24092100185@cytiu.edu.mo (H.Y.); 2 Shenzhen Institute of Advanced Technology, Chinese Academy of Sciences, Shenzhen 518055, China

**Keywords:** dexterous grasping, conditional diffusion, grasp generation, diffusion policy, receding-horizon execution

## Abstract

Dexterous grasping with multi-finger robotic hands is essential for general-purpose robotic manipulation, but remains challenging due to high-dimensional hand configurations, multimodal grasp distributions, and contact-rich execution dynamics. Existing methods often decouple grasp target generation from execution policy learning, which limits the consistency between generated grasp goals and downstream control. To address this problem, we propose DexGraspDiffuser, a target-coupled grasp and action diffusion framework for dexterous grasping. The first stage, GraspDiffusion, generates diverse and physically plausible target grasps from object point clouds using a compact representation of hand root translation, continuous rotation, and finger joint configuration. The second stage, a Goal-Conditioned Diffusion Policy, predicts temporally coherent action sequences conditioned on the selected target grasp and current observation. During inference, receding-horizon execution enables action-prefix execution and online replanning for improved robustness. Experiments demonstrate that DexGraspDiffuser achieves success rates of 0.76, 0.72, and 0.68 on training objects, unseen objects from seen categories, and objects from unseen categories, respectively. These results correspond to a three-split average success rate of 0.72 and a train-to-unseen generalization gap of 0.08. Compared with the reproduced UniDexGrasp-T baseline under the same object split and evaluation protocol, DexGraspDiffuser improves the three-split average success rate by 3.3 percentage points and reduces the average mean position error by 0.53 cm. This indicates that target-coupled grasp and action diffusion contribute to improved grasp quality, execution accuracy, and closed-loop stability.

## 1. Introduction

Dexterous grasping is a fundamental capability for robots to manipulate complex objects, with broad applications in service robotics, intelligent manufacturing, and warehouse sorting [1]. Compared with two-finger grippers, multi-finger dexterous hands can establish richer interactions with objects through the palm, fingertips, and multiple contact regions [2,3]. This makes them better suited for diverse grasping and manipulation tasks, such as enveloping grasps, lateral grasps, and fine manipulation [4]. However, the increased dexterity also brings substantial modeling challenges. Five-finger dexterous hands, such as the ShadowHand, have high-dimensional joint spaces [5]. As a result, dexterous grasping is no longer limited to estimating the pose of an end-effector; it must jointly account for the palm pose, finger joint configuration, object geometry, contact relationships, and the subsequent motion required for execution [6,7,8,9]. Therefore, grasping methods for dexterous hands should not only identify feasible grasp targets, but also generate continuous control sequences that can reliably reach the target, establish stable contacts, and lift the object.

Existing studies typically address target generation and execution control in high-degree-of-freedom dexterous grasping at two levels. Grasp generation methods predict feasible hand configurations from object geometry, whereas grasp execution methods learn control policies that drive the hand from the current state toward a target configuration. Therefore, decomposing dexterous grasping into two stages, namely grasp target generation and target-conditioned execution, can reduce the modeling complexity of high-dimensional manipulation tasks [10] and establish a clear interface between geometric perception and motion control [11], yet this framework still faces two key challenges. First, dexterous grasp targets are often highly multimodal. The same object may admit multiple valid hand root poses and joint configurations, while simple regression models tend to produce averaged predictions with limited physical plausibility [12]. Second, successful execution depends not only on the target grasp itself, but also on the coordinated use of object point clouds, robot body state, hand state, and real-time observations to continuously adjust actions during contact [13,14].

Diffusion models provide a suitable generative framework for addressing both challenges. Unlike direct regression, diffusion models learn the underlying data distribution through progressive noise injection and reverse denoising, which allows them to represent multimodal output spaces more effectively [15,16]. For grasp target generation, this enables the model to produce diverse candidate grasps conditioned on object point clouds, rather than predicting a single deterministic hand configuration [17]. Recent studies have applied diffusion models to high-dimensional dexterous grasp generation, showing their ability to model complex hand configurations while accounting for geometric and physical feasibility constraints [18,19]. For execution control, diffusion policies formulate policy learning as conditional action-sequence generation, predicting an entire future action horizon instead of outputting individual control commands step by step [20,21,22,23]. This trajectory-level formulation better matches the sequential structure of dexterous grasping, including approach, pre-shaping, contact formation, and closure. However, existing methods mainly focus on static grasp targets, whereas action diffusion policies are typically designed for general manipulation tasks. These two lines of work have not yet been fully integrated into a unified framework for target-conditioned dexterous grasping. Therefore, a key challenge is to effectively connect grasp target generation, target-conditioned action sequence generation, and closed-loop execution, thereby improving the stability and robustness of high-degree-of-freedom dexterous grasping.

Recent universal dexterous grasping methods have improved generalization through structured policy learning, scalable trajectory supervision, and task-level priors. UniDexGrasp++ introduces geometry-aware curriculum learning and iterative generalist-specialist learning for large-scale goal-conditioned grasping [24]. UniGraspTransformer distills successful trajectories from object-specific reinforcement learning policies into a universal Transformer-based policy [25], while AffordDex incorporates human motion priors and negative affordance-aware reasoning to improve functional appropriateness and human-likeness [26]. Complementary to these large-scale or task-prior-driven systems, this paper focuses on a practical target-conditioned setting that couples grasp target generation, action-sequence diffusion, and closed-loop replanning under a moderate expert-trajectory budget.

Motivated by these observations, we propose DexGraspDiffuser, a target-coupled conditional diffusion framework for practical goal-conditioned dexterous grasping. The framework formulates grasp target generation and goal-conditioned action execution as two coupled conditional generation problems. In the first stage, GraspDiffusion generates target grasps from object point clouds in a compact representation space consisting of hand root translation, continuous rotation, and finger joint configuration. In the second stage, a goal-conditioned diffusion policy generates temporally consistent local action horizons conditioned on the current observation and selected target grasp. The target grasp serves as an explicit interface between geometric perception and action execution, enabling the policy to generate local trajectories under a specified grasp intention. During inference, receding-horizon execution is adopted to execute action prefixes and replan with updated observations, improving robustness against contact disturbances, observation noise, and action errors.

The main contributions of this paper are as follows:

- We propose DexGraspDiffuser, a target-coupled two-stage conditional diffusion framework that connects point-cloud-based grasp target generation with goal-conditioned dexterous execution.
- We design GraspDiffusion to model multimodal target grasp distributions in a compact grasp representation space, providing feasible candidate goals for downstream execution.
- We introduce a goal-conditioned diffusion policy that generates temporally consistent local action horizons conditioned on point cloud observations, robot states, and selected target grasps.
- We incorporate receding-horizon diffusion execution to enable action-prefix execution and online replanning, improving robustness under contact disturbances, observation noise, and action deviations.

## 2. Related Work

### 2.1. Dexterous Grasp Target Generation

Grasp target generation aims to predict executable hand target configurations from object geometry and provide stable conditions for subsequent execution. Early methods mainly used analytical models, contact optimization, or simulation tools, such as GraspIt! for collision detection and grasp quality evaluation [27], force-closure planning for humanoid robot hands [28], ContactDB for learning hand–object contact patterns [4], and ContactOpt for contact-based pose refinement [7]. These methods laid the foundation for learning-based grasp synthesis, but often rely on strong manual priors or carefully designed objectives when handling complex objects, multi-category generalization, and high-dimensional hand configurations. Recent learning-based methods are commonly categorized into sampling-based generation, direct regression, reinforcement learning, and example retrieval [29]. Recent studies have also explored grasp pose prediction from motion priors [30] and dexterous grasp synthesis with fine-grained contact generation and natural pose optimization [31]. Contact-GraspNet and AnyGrasp focus on 6-DoF grasping from point clouds or depth observations [32,33]. For dexterous hands, UniDexGrasp formulates grasping as a goal-conditioned problem by combining grasp proposal generation with goal-conditioned execution [9]. UniDexGrasp++ further improves this paradigm through geometry-aware curriculum learning and iterative generalist-specialist learning, enhancing policy generalization across large-scale object sets [24]. RealDex, G-DexGrasp, and D(R,O)Grasp extend dexterous grasp generation by incorporating human grasp priors, component-level prior retrieval, and cross-embodiment hand–object interaction representations [34,35,36].

Although these methods have advanced grasp target generation, a static hand configuration alone is insufficient for reliable execution with high-degree-of-freedom dexterous hands. Existing methods often insufficiently model multi-joint configurations and complex hand–object contacts, and give limited attention to how generated targets are coupled with downstream control. In target-conditioned dexterous grasping, the grasp target should act not only as a geometric prediction, but also as an intermediate condition linking point-cloud perception and action execution. Therefore, this paper formulates grasp target generation as a point-cloud-conditioned diffusion process and passes the generated target to a target-conditioned action policy, enabling grasp generation to support the full execution loop.

### 2.2. Applications of Diffusion Models in Grasp Generation and Action Policies

Diffusion models have recently become effective tools for complex generative tasks. DDPM learns data distributions through progressive noise addition and reverse denoising, offering a stable framework for generation in high-dimensional continuous spaces [15]. This property is well-suited to robotic grasping and action generation, where both grasp targets and action trajectories are often multimodal. For grasp generation, recent works have explored diffusion-related generative modeling for contact map refinement and dexterous grasp synthesis [31], while DexGrasp Anything integrates physical constraints into the diffusion process to improve grasp feasibility and stability [19]. For policy learning, Diffusion Policy formulates visuomotor control as conditional denoising and generates action chunks for continuous manipulation [12,14]. Related methods, including Equivariant Diffusion Policy [37], ACT [38], PerAct [39], MimicGen [40], and RoboMimic [41], further improve trajectory-level policy learning from the perspectives of symmetry, action chunking, 3D representations, demonstration generation, and offline imitation learning.

Existing diffusion-based grasp generation methods and diffusion-based action policies have shown promising progress, but their coupling remains limited in target-conditioned dexterous grasping tasks. Grasp diffusion methods mainly focus on static target generation, whereas action diffusion policies are typically designed for general manipulation tasks and do not explicitly model the conditional relationships among target grasps, hand states, and contact execution. To address this limitation, this paper integrates grasp target generation and action sequence generation into a target-coupled conditional diffusion framework. The generated grasp target is used to directly condition subsequent action planning, thereby improving execution consistency.

### 2.3. Multi-Source Conditional Information and Closed-Loop Execution

The execution phase of dexterous grasping requires the joint processing of object geometry, robot proprioceptive state, hand state, and target grasp information [2,13]. Object point clouds describe the spatial structure of the object; proprioceptive and hand states characterize the current execution context; and the target grasp defines the hand configuration toward which the policy should converge. Compared with using only a single-frame geometric observation, incorporating these multi-source conditions enables the policy to better infer the current hand–object relationship and generate target-consistent actions. Related reviews suggest that the performance of robotic grasping and dexterous manipulation depends not only on grasp generation but also on the coordination of perception, state estimation, feedback control, and execution policies [42,43]. Recent universal dexterous grasping systems further support this view. UniGraspTransformer shows that scalable dexterous grasping can benefit from trajectory-level supervision and high-capacity Transformer policies distilled from object-specific reinforcement learning policies [25]. AffordDex further demonstrates that human motion priors and negative affordance-aware reasoning can improve the functional quality and human-likeness of dexterous execution [26]. These studies indicate that robust dexterous execution depends not only on feasible target grasps, but also on how object geometry, robot state, hand state, trajectory supervision, and task-related priors are organized during policy learning. In contact-rich manipulation, vision, tactile sensing, and proprioception are often integrated to improve contact state estimation and operational stability. Recent tactile works such as NeuralFeels [44], 3D-ViTac [45], and Sparsh [46] further demonstrate the importance of multi-source perception for fine-grained manipulation and contact-intensive tasks [47,48]. Beyond tactile representation learning, recent sensor-level studies have explored flexible multimodal sensing for robotic grasping. For example, Dong et al. developed a machine learning-assisted flexible dual-modal electronic-skin sensor that jointly perceives proximity distance and contact pressure, supporting material and hardness recognition during grasping [49]. While such contact and proximity feedback improves grasp perception, DexGraspDiffuser focuses on target-coupled grasp and action generation from point-cloud and proprioceptive observations. Thus, these sensing methods are complementary to our framework and could be integrated in future work to enhance contact-state estimation and sim-to-real transfer.

However, existing dexterous grasp execution methods still underutilize multi-source conditional information and closed-loop correction, which makes them susceptible to error accumulation under contact disturbances, observation noise, and action deviations. Recent large-scale dexterous grasping systems further demonstrate the importance of structured policy learning, scalable trajectory supervision, and task-level priors. UniDexGrasp++ improves universal dexterous grasping through geometry-aware curriculum learning and iterative generalist–specialist policy training. UniGraspTransformer distills successful trajectories from object-specific reinforcement learning policies into a universal Transformer-based policy, showing that scalable trajectory supervision and high-capacity architectures can improve generalization in dexterous grasping. AffordDex further introduces human motion priors and negative affordance-aware reasoning to improve functional contact appropriateness and human likeness. Nevertheless, these benefits often come with more complex training pipelines, larger-scale interaction data, high-capacity policy models, or additional task-level priors. DexGraspDiffuser addresses a complementary target-conditioned setting by coupling point-cloud-conditioned grasp target generation with goal-conditioned action-sequence diffusion under a moderate expert-trajectory budget. Together with receding-horizon execution, this design provides a computationally accessible route toward robust and generalizable dexterous grasping in simulation, while remaining compatible with future extensions such as curriculum learning, trajectory distillation, Transformer-based policies, and affordance-aware priors. Recent large-scale methods such as UniDexGrasp++ and UniGraspTransformer further demonstrate the value of curriculum learning, object-specific reinforcement learning, trajectory distillation, and high-capacity policies [24,25]. Specifically, UniDexGrasp++ improves universal dexterous grasping through geometry-aware curriculum learning and iterative generalist–specialist policy training, while UniGraspTransformer distills successful trajectories from object-specific reinforcement learning policies into a universal Transformer-based policy. AffordDex further introduces a two-stage affordance-aware dexterous grasping framework that combines human motion priors with negative affordance-aware reasoning, aiming to improve not only grasp success but also human-like motion and functional contact appropriateness [26]. Nevertheless, these benefits often come with substantial simulator interaction, large trajectory sets, high-capacity policy architectures, additional task-level priors, or complex training pipelines. DexGraspDiffuser addresses a complementary goal-conditioned setting by coupling point-cloud-conditioned grasp target generation with goal-conditioned action-sequence diffusion under a moderate expert-trajectory budget. Together with receding-horizon execution, this design provides a computationally accessible route toward robust and generalizable dexterous grasping in simulation, while remaining compatible with future extensions such as curriculum learning, trajectory distillation, Transformer-based policies, and affordance-aware priors.

## 3. Methodology

This section presents DexGraspDiffuser, a two-stage conditional diffusion framework for dexterous grasping. The method first models grasp targets from object point clouds and then generates goal-conditioned action sequences for execution. By coupling grasp proposal generation with receding-horizon execution (RHC), DexGraspDiffuser improves the diversity and feasibility of grasp targets while enhancing execution robustness under contact-rich interactions.

### 3.1. Problem Formulation

This paper proposes DexGraspDiffuser, a two-stage conditional diffusion framework for goal coupling. Given an object point cloud $X_{0}$, the framework first predicts an executable grasping target *g*, and then generates an action sequence conditioned on the current observation $o_{t}$. The generated actions are executed in the RHC loop. This design enables a dexterous hand to progressively lift an object from its current state. The task consists of two closely related components: geometric goal modeling in the grasping space and temporal action generation in the control space. The former specifies the desired hand–object configuration, while the latter realizes this configuration through continuous interaction. Dexterous grasping is therefore formulated as a target-coupled conditional generation process:

(1) $$p\left(g,A_{t}|X_{0},o_{t}\right)=p_{\theta }\left(g|X_{0}\right)\cdot p_{\omega }\left(A_{t}|o_{t},g\right)$$

where $p(g|X_{0})$ denotes the grasp target distribution and $p_{\omega }\left(A_{t}|o_{t},g\right)$ denotes the goal-conditioned action sequence distribution. $A_{t}=\left[a_{t},\ldots ,a_{t+H-1}\right]$ represents the local action sequence predicted at time *t*, and *H* represents the action prediction length. The parameters θ and ω correspond to the GraspDiffusion model and the goal-conditional diffusion policy, respectively.

Equation (1) formulates target selection and temporal execution control as two coupled conditional distributions. The grasp target *g* encodes geometric grasp intent from the object point cloud and provides an explicit condition for action generation, thereby reducing the decision complexity in the high-dimensional dexterous hand action space.

**Figure 1:**
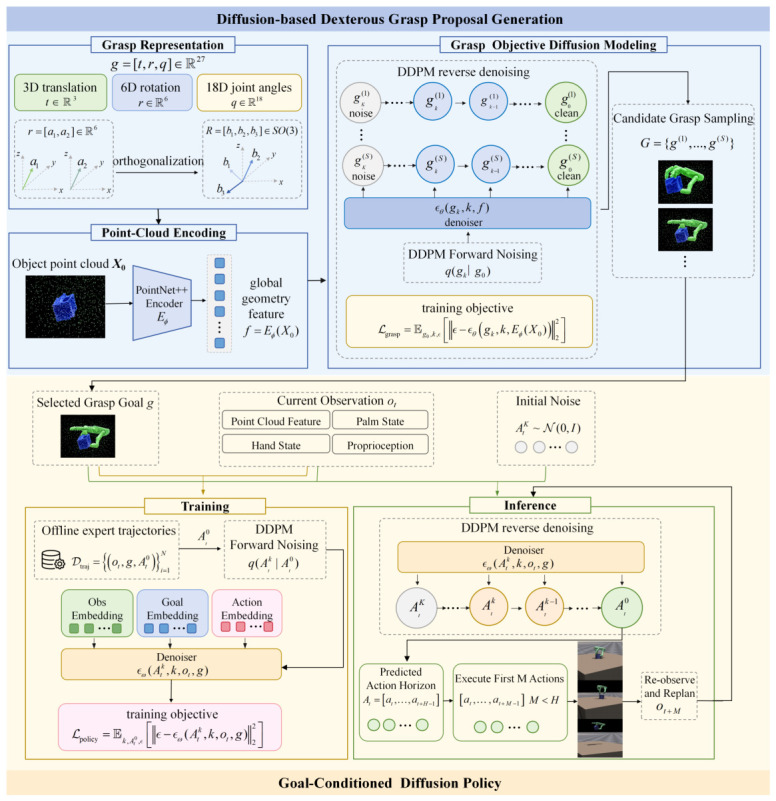
The pipeline of DexGraspDiffuser.

Figure 1 illustrates the pipeline of DexGraspDiffuser. A GraspDiffusion model samples candidate grasp targets from the object point cloud, and a goal-conditioned diffusion policy predicts short-horizon actions conditioned on the selected target and current observation. During inference, only the first *M* actions are executed before replanning with updated observations, enabling closed-loop dexterous grasping in a receding-horizon manner.

### 3.2. Diffusion-Based Dexterous Grasp Proposal Generation

#### 3.2.1. Grasp Representation and Point Cloud Encoding

The first stage models the distribution of feasible target grasps conditioned on object geometry. A target grasp is represented as

(2) $$g=\left[t,r,q\right]\in R^{27}$$

where $t\in R^{3}$ denotes the hand root translation, $r\in R^{6}$ denotes the continuous 6D representation of the hand root rotation, and $q\in R^{18}$ denotes the finger joint configuration. Given $r=[a_{1},a_{2}]$, $a_{1},a_{2}\in R^{3}$, the rotation matrix $R\left(r\right)=\left[b_{1},b_{2},b_{3}\right]\in SO\left(3\right)$ is obtained through orthogonalization. This representation embeds the dexterous grasping mechanism into Euclidean space, enabling its modeling with standard diffusion models. We adopt the continuous 6D rotation representation rather than Euler angles or quaternions because it is more suitable for diffusion-based grasp generation in a continuous Euclidean space. Euler angles provide a compact three-dimensional representation, but they suffer from discontinuities and gimbal-lock issues, which may introduce unstable targets during denoising. Quaternions avoid gimbal lock, but they require unit-norm normalization and have a double-cover ambiguity, where two antipodal quaternion representations correspond to the same rotation. These constraints and ambiguities are not fully aligned with the unconstrained Gaussian perturbation and Euclidean noise-prediction objective used in DDPM. In contrast, the 6D representation can be directly perturbed and denoised in Euclidean space and then mapped to SO(3) through orthogonalization. Although it introduces two additional dimensions compared with quaternions, this overhead is small relative to the full 27-dimensional grasp vector and provides better continuity and numerical stability for grasp target generation.

To ensure that the captured data conforms to object geometry constraints, we first input the point cloud $X={\left\{x_{i}\right\}}_{i=1}^{N},x_{i}\in R^{3}$ [50], and the object point cloud $X_{0}$ is encoded by a PointNet++ encoder [51]:

(3) $$f=E_{\phi }\left(X_{0}\right)$$

where *f* is the global object geometry feature. The encoder aggregates local shape, global scale information, and spatial structure from the unordered point set. GraspDiffusion performs denoising generation based on *f*, ensuring that the generated target grasp is consistent with the observed object geometry.

#### 3.2.2. Conditional Diffusion over Grasp Targets

The nature of dexterous grasping is multimodal. For the same object, valid grasps may involve different contact regions, leading to distinct palm positions, palm orientations, and finger configurations. If a deterministic mapping X→g is learned directly, the model often tends to predict the conditional mean, which may not correspond to any physically feasible grasp. To address this issue, we adopt a conditional denoising diffusion model to directly learn the conditional distribution $p_{\theta }\left(g|X\right)$ over the full grasp vector.

Both diffusion modules in DexGraspDiffuser adopt the standard DDPM noise-prediction formulation. For a clean sample $x_{0}$, the forward diffusion process is

(4) $$x_{k}=\sqrt{{\bar{\alpha }}_{k}}x_{0}+\sqrt{1-{\bar{\alpha }}_{k}}\epsilon ,\;\epsilon ∼N\left(0,I\right)$$

where *k* is the diffusion step and ${\bar{\alpha }}_{k}$ is determined by the noise schedule. In the grasp generation stage, $x_{0}$ corresponds to the real target grasp $g_{0}$. The denoising network $\epsilon_{\theta }\left(g_{k},k,f\right)$ predicts the injected noise from the noisy grasp $g_{k}$, diffusion step *k*, and geometry condition *f*. The training objective is

(5) $$L_{\mathrm{grasp}}=E_{g_{0},k,\epsilon }\left[{∥\epsilon -\epsilon_{\theta }\left(g_{k},k,E_{\phi }\left(X_{0}\right)\right)∥}_{2}^{2}\right]$$

This objective drives the model to learn the denoising direction of the object-conditioned grasp distribution. At high noise levels, the denoising process recovers coarse spatial relations between the hand and the object, such as the approach side and approximate root orientation. At low noise levels, it refines the rotation and joint configuration to form a physically meaningful grasp target. Therefore, the progressive denoising process provides a natural mechanism for modeling the multi-peak structure of dexterous grasp targets.

During the inference stage, GraspDiffusion samples *S* candidate target grasps from Gaussian noise:

(6) $$G=\left\{g^{\left(1\right)},g^{\left(2\right)},\ldots ,g^{\left(S\right)}\right\}$$

where *g* is the target grasp proposal. The sampled candidates are ranked according to grasp quality, penetration depth, and collision validity. The selected target grasp is then passed to the execution policy as the goal condition. This candidate generation and selection process expands the search space of feasible target grasps while ensuring that the target provided to the policy is geometrically valid and executable.

### 3.3. Goal-Conditioned Diffusion Policy for Dexterous Execution

#### 3.3.1. Expert Trajectory and Action Sequence Representation

The first-stage grasp target *g* specifies only the desired final hand configuration, not the the trajectory required to reach it. Since dexterous grasping involves approach, pre-shaping, contact formation, and lift, we formulate execution as goal-conditioned trajectory generation rather than single-step motion regression. We represent the policy output as a local action sequence:

(7) $$A_{t}^{0}=\left[a_{t},a_{t+1},\ldots ,a_{t+H-1}\right]$$

where $A_{t}^{0}$ is the expert action sequence and *H* is the action prediction length. Action sequence modeling enables policies to characterize the cooperative relationships of actions within a local time window, thereby better maintaining the continuity between different processes.

We construct an offline expert trajectory dataset with a goal-conditioned teacher policy [9] and retain successful trajectories to provide high-quality supervision for the diffusion policy. From each successful trajectory, we extract local action windows of length *H* using a sliding window, forming the offline trajectory dataset:

(8) $$D_{\mathrm{traj}}={\left\{\left(o_{t},g,A_{t}^{0}\right)\right\}}_{i=1}^{N}$$

where $o_{t}$ represents the current observation, including point-cloud feature, hand state, palm pose, and proprioceptive state. The diffusion policy learns successful action distributions from these trajectories and generates action sequences conditioned on current observations and the selected target at inference. We use 50 successful expert trajectories per object as the default setting for constructing $D_{\mathrm{traj}}$; for detailed analysis, please refer to Section 4.1.

#### 3.3.2. Conditional Action Sequence Diffusion

This paper employs an action diffusion policy to model the distribution of local action sequences. Given a clean expert action sequence $A_{t}^{0}$, the forward process in Equation (4) produces a noisy action sequence $A_{t}^{k}$. The denoising network $\epsilon_{\omega }\left(A_{t}^{k},k,o_{t},g\right)$ predicts the noise conditioned on the noisy action sequence, diffusion step, current observation, and target grasp. The policy training objective is

(9) $$L_{\mathrm{policy}}=E_{A_{t}^{0},k,\epsilon }\left[{∥\epsilon -\epsilon_{\omega }\left(A_{t}^{k},k,o_{t},g\right)∥}_{2}^{2}\right]$$

Equation (9) determines the execution characteristics of the second stage. Based on the current observation $o_{t}$ and the target grasp *g*, the diffusion policy learns the conditional distribution, keeping action generation aligned with the current execution and the selected grasp target.

#### 3.3.3. Receding-Horizon Execution

During inference, the goal-conditioned diffusion policy generates a short-horizon action sequence at each control step. Because object poses, contact states, and point-cloud observations may change after interaction, dexterous grasping requires continuous correction. We therefore execute the policy in a receding-horizon manner, enabling closed-loop feedback throughout the grasping process.

At time *t*, the policy generates an action sequence, $A_{t}=\left[a_{t},\ldots ,a_{t+H-1}\right]$ based on the current observations and target grasp, the controller executes only the first *M* actions $\left[a_{t},\ldots ,a_{t+M-1}\right],M<H$. After these actions are applied, the system receives an updated observation $o_{t+M}$ and generates the next action horizon:

(10) $$A_{t+M}∼p_{\omega }\left(A|o_{t+M},g\right)$$

This procedure turns the diffusion policy into a closed-loop controller. The prediction horizon ensures local temporal consistency, while recurrent observation and replanning enable adaptation to contact-induced deviations, which is critical for robust contact-rich manipulation.

To improve action continuity between successive replanning windows, we apply exponential smoothing in the RHC setting:

(11) $${\tilde{a}}_{t}=\left(1-\rho \right)a_{t}+\rho {\tilde{a}}_{t-1}$$

where ρ is the smoothing coefficient. The smoothing term reduces discontinuities at the boundary of adjacent horizons and improves low-level execution stability.

### 3.4. Training and Inference

DexGraspDiffuser separately trains two conditional diffusion modules. GraspDiffusion learns the target grasp distribution from static grasp labels $\left(X_{0},g\right)$ conditioned on the object point cloud, while the Goal-Conditioned Diffusion Policy learns the local action-sequence distribution from successful expert trajectory windows $\left(o_{t},g,A_{t}^{0}\right)$ conditioned on the target grasp. During inference, the two modules are coupled through the selected target grasp. The complete inference procedure is as follows.

(12) $$\begin{array}{cc} \; & f=E_{\phi }\left(X_{t}\right),\\ \; & G={\left\{g^{\left(s\right)}\right\}}_{s=1}^{S}∼p_{\theta }\left(g|X_{t}\right),\\ \; & g=\mathrm{Select}(G),\\ \; & A_{t}∼p_{\omega }\left(A_{t}|o_{t},g\right),\\ \; & \mathrm{execute}\left[a_{t},\ldots ,a_{t+M-1}\right],\;t\leftarrow t+M. \end{array}$$

This process first constructs a target grasp distribution from object geometry, uses the selected target as a condition for local action diffusion, and performs execution and replanning in an RHC manner. Using the target grasp as a shared condition, DexGraspDiffuser integrates target generation conditioned on point clouds, action diffusion conditioned on target grasps, and closed-loop execution into a unified dexterous grasping framework. This design enables the model to capture multimodal target distributions, temporal action structures, and uncertainties during contact-rich execution.

## 4. Experimental Results and Analysis

### 4.1. Experimental Setup

We evaluate DexGraspDiffuser in a goal-conditioned dexterous grasping simulation using ShadowHand as the dexterous hand actuator, and conduct our experiments in Isaac Gym [52] simulator. The model is tested in a desktop grasping scenario under three object settings: training objects, unseen objects from seen categories, and objects from unseen categories. For a fair comparison, we adopt the same object split and evaluation protocol as Ref. [9].

**Figure 2:**
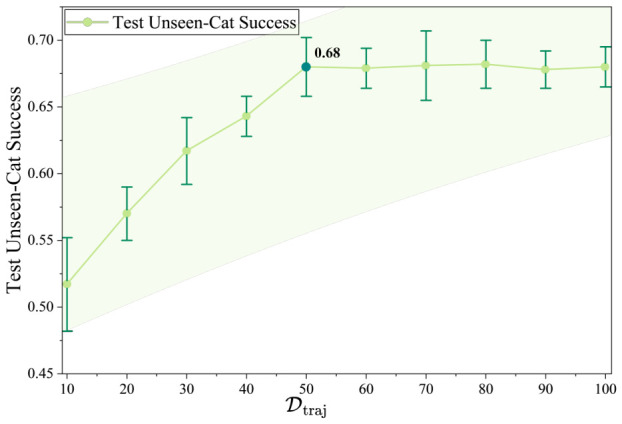
Sensitivity analysis on the number of expert trajectories per object.

The dataset comprises approximately 1.12 million valid grasps across 5519 object instances from 133 object categories. We split the object instances into 3251 for training, 754 for testing on unseen objects from seen categories, and 1514 for testing on objects from unseen categories. The first stage, the GraspDiffusion is trained with static grasp labels $\left(X_{0},g\right)$, while the second stage, the Goal-Conditioned Diffusion Policy is trained using action sequences extracted from successful expert trajectories. For reproducibility, we further specify the filtering criteria used to retain successful expert trajectories. Each expert rollout is executed for a maximum episode length of 250 control steps. A trajectory is regarded as successful only if the object center is lifted by at least 0.10 m relative to its initial height and remains above this threshold for at least 20 consecutive control steps. Trajectories are discarded if the object is dropped after lifting, leaves the workspace, exhibits invalid simulation states such as NaN states or exploding velocities, or violates the physical validity constraint with severe hand–object penetration. In our implementation, severe penetration is defined as a maximum penetration depth larger than 1 cm for more than five consecutive frames. We also require stable hand–object interaction during the final stability window, with at least two valid hand–object contact regions. For each object, we retain 50 successful expert trajectories using a fixed random seed. If more than 50 successful trajectories are available, 50 trajectories are sampled without replacement; if fewer than 50 are available, all valid successful trajectories are retained. From each retained trajectory, local action windows of length H=16 are extracted using a sliding-window stride of 1, and incomplete terminal windows are discarded. We further analyze the sensitivity to the number of expert trajectories per object. Since unseen-category objects better reflect policy generalization, we report the success rate on the unseen-category test set. As shown in Figure 2, performance improves rapidly from 10 to 50 trajectories, but becomes nearly saturated after 50 trajectories and remains around 0.68. Therefore, we use 50 expert trajectories per object as the default setting, which provides a reasonable trade-off between data scale and execution performance.

All experiments are conducted on a workstation equipped with an RTX 4090 GPU with 24 GB of VRAM, a 32-core CPU, and 224 GB of RAM. The GPU provides approximately 83 TFLOPs of peak FP32 performance. Except for the deterministic motion-planning baseline, MP, all learning-based methods are trained with three independent random seeds and evaluated using the same object split and evaluation protocol. Results are reported as mean ± standard deviation. The UniDexGrasp-T and UniDexGrasp-S baselines are independently reproduced following the original UniDexGrasp protocol, including the two-stage pipeline of grasp proposal generation and goal-conditioned grasp execution. Specifically, we follow the original teacher–student policy learning procedure, where a state-based teacher policy is first trained and then distilled into a vision-based student policy. Since the reproduction is conducted under our local implementation environment with independently selected random seeds, small numerical differences from the originally reported values may occur. The main training hyperparameters of DexGraspDiffuser are summarized in Table 1.

### 4.2. Overall Grasping Performance

This experiment assesses the end-to-end performance of DexGraspDiffuser within a complete dexterous grasping pipeline. We compare our method with MP, PPO [53], DAPG [54], ILAD [55], and the teacher and student policies of UniDexGrasp [9] under the same evaluation protocol. By testing on training objects, unseen instances from seen categories, and objects from unseen categories, it provides a direct evaluation of the method’s overall grasp success rate and execution accuracy.

As shown in Table 2, DexGraspDiffuser achieves the highest success rates across the Train, Test Seen-Cat, and Test Unseen-Cat splits, with scores of 0.76 ± 0.016, 0.72 ± 0.019, and 0.68 ± 0.022, respectively. Compared with the UniDexGrasp Student, our method improves the success rate on unseen-category objects from 0.58 ± 0.026 to 0.68 ± 0.022. This result suggests that the proposed two-stage conditional diffusion framework improves generalization to objects from unseen categories. This improvement stems from Diffusion Policy’s ability to learn complex multi-finger cooperative action distributions from expert demonstrations, reducing the exploration and convergence challenges associated with reinforcement learning in sparse-reward settings. DexGraspDiffuser also consistently outperforms the UniDexGrasp Teacher across all three splits, indicating that diffusion-based target generation and target-conditioned action sequence modeling can not only replace the original execution policy, but also further improve end-to-end grasping performance.

In terms of execution accuracy, DexGraspDiffuser obtains the lowest MPE among all learning-based methods. On the Test Unseen-Cat split, it achieves an MPE of 4.0 ± 0.25 cm, compared with 4.7 ± 0.31 cm for UniDexGrasp Teacher and 4.9 ± 0.34 cm for UniDexGrasp Student. These results indicate that the goal-conditioned diffusion policy can execute the generated grasp targets more accurately. Although MP obtains a relatively low MPE, its success rate is substantially lower, especially on unseen-category objects, where it reaches only 0.02 ± 0.005. Therefore, its low MPE does not translate into effective end-to-end dexterous grasping performance. To avoid overestimating the improvement by averaging across weak or less comparable baselines, we report the improvement relative to the strongest directly comparable reproduced baseline, UniDexGrasp-T. Across the three evaluation splits, DexGraspDiffuser achieves a 3.3 percentage-point improvement in average success rate over UniDexGrasp-T and reduces the average mean position error by 0.53 cm.

**Figure 3:**
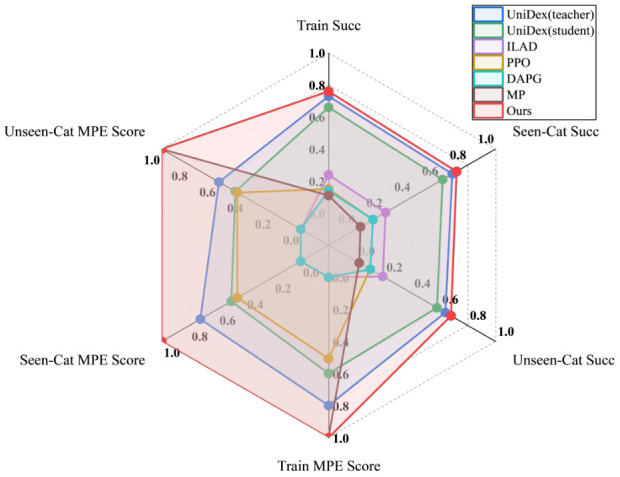
Comparison of grasp performance of different models in radar map.

To further compare the overall performance of different methods in terms of success rate and execution accuracy, we visualize the success rate and MPE results using a radar chart, as shown in Figure 3. Since higher success rates indicate better performance, whereas lower MPE values are preferred, we convert MPE into a normalized MPE score so that all radar-chart axes have the same direction. For each data split s∈{Train,Seen−Cat,Unseen−Cat}, the MPE score is defined as

(13) $$\mathrm{MPE}\;{\mathrm{Score}}_{m}^{s}=1-\frac{{\mathrm{MPE}}_{m}^{s}-\min_{j\in M}{\mathrm{MPE}}_{j}^{s}}{\max_{j\in M}{\mathrm{MPE}}_{j}^{s}-\min_{j\in M}{\mathrm{MPE}}_{j}^{s}+\epsilon }$$

where *m* represents the method to be evaluated, M represents the set of methods participating in the normalization, and ϵ is a minimal constant to prevent the denominator from being zero. After this transformation, the smaller the MPE, the higher the corresponding MPE score.

Figure 3 shows that DexGraspDiffuser consistently reaches the outermost region on the Train Succ, Seen-Cat Succ, and Unseen-Cat Succ, indicating higher grasping success rates across all evaluation settings. It also preserves strong performance across the three MPE score dimensions, suggesting that the goal-conditioned diffusion policy can accurately execute the grasp targets generated in the first stage. These observations are consistent with the quantitative results in Table 2 and show that DexGraspDiffuser achieves a favorable balance between end-to-end success rate and target execution accuracy.

**Figure 4:**
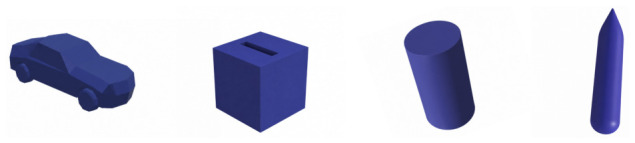
Representative objects for simulation visualization.

Figure 4 presents representative objects used for simulation visualization in this study, including a vehicle, a tissue box, a cylinder, and a pencil-like slender object. These objects differ markedly in geometry. The vehicle-shaped object contains irregular contours and local protrusions, the box-shaped object exhibits clear planar surfaces and sharp edges, the cylinder is dominated by curved surfaces, and the slender object requires more precise grasp posture and finger closure. Together, these objects cover a range of geometric characteristics and provide a qualitative basis for demonstrating the model’s grasp generation and execution capability under different shape conditions.

**Figure 5:**
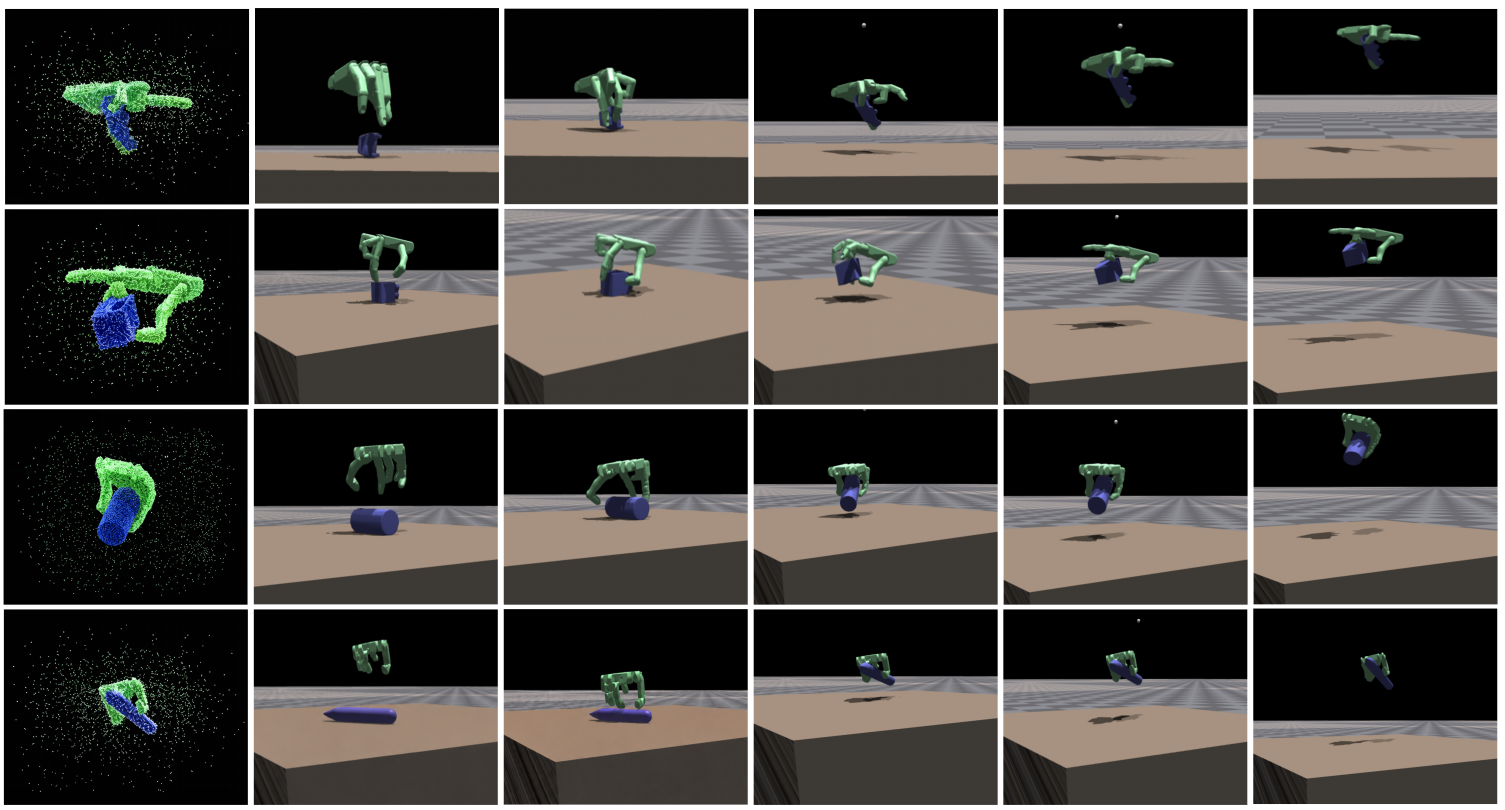
Qualitative grasping simulation results.

Figure 5 illustrates the simulated grasping process of DexGraspDiffuser on different object categories. Each row shows one object type, including the point-cloud observation, the generated target grasp configuration, and the subsequent execution process driven by the goal-conditioned diffusion policy. The visualizations show that DexGraspDiffuser adapts the grasp target to object geometry and guides the ShadowHand through approach, pre-shaping, contact, and lifting. It produces stable lateral or enveloping grasps for box-shaped and cylindrical objects, effective contacts along the long axis of slender objects, and suitable palm orientations and finger configurations for irregular vehicle-shaped objects.

Overall, the simulation visualization results are consistent with the dexterous grasping results reported above. GraspDiffusion generates feasible target grasps for objects with different geometries, and the goal-conditioned diffusion policy produces continuous action sequences conditioned on the target grasp to complete the execution process. These results suggest that the proposed method not only achieves improved success rates and lower error metrics, but also exhibits good geometric adaptability and execution stability during grasping.

Although the quantitative comparisons in Table 2 focus on methods that can be reproduced and evaluated under the same object split and goal-conditioned dexterous grasping protocol, recent large-scale methods such as UniDexGrasp++ and UniGraspTransformer provide important complementary references for understanding the trade-off between training scale and framework accessibility [24,25]. UniDexGrasp++ emphasizes large-scale generalization through geometry-aware curriculum learning and iterative generalist–specialist training, whereas UniGraspTransformer relies on trajectory distillation from object-specific reinforcement learning policies and high-capacity Transformer-based policy learning. AffordDex further introduces affordance-aware dexterous grasping by combining human motion priors with negative affordance-aware reasoning, thereby improving functional appropriateness and human likeness [26]. These methods are powerful large-scale systems, but they generally require substantial simulator interaction, large trajectory sets, high-capacity architectures, or additional task-level priors.

In contrast, DexGraspDiffuser focuses on a lightweight goal-conditioned setting that explicitly couples point-cloud-conditioned grasp target diffusion with goal-conditioned action-sequence diffusion under a moderate expert-trajectory budget. Its main advantage lies in target-action consistency, diffusion-based temporal action modeling, closed-loop receding-horizon correction, and relatively accessible training requirements. Therefore, DexGraspDiffuser should be viewed as complementary to UniDexGrasp++, UniGraspTransformer, and AffordDex rather than as a direct replacement for large-scale universal or affordance-aware dexterous grasping systems. Future work may integrate curriculum learning, trajectory distillation, Transformer-based policy architectures, or affordance-aware priors into the proposed target-coupled diffusion framework to further improve large-scale generalization and functional grasp quality.

### 4.3. Grasp Proposal Generation Evaluation

Since end-to-end grasp performance depends not only on the execution policy but also on the quality of the initial grasp generation proposals, we separately evaluate the proposal generation module. We focus on whether the generated candidates are physically feasible, geometrically plausible, and sufficiently diverse. The evaluation metrics used in this experiment are shown in Table 3. We compare GraspDiffusion with GraspTTA [56], DDG [57], ReLie [58], ProHMR [59], and UniDexGrasp [9] under the same grasp proposal evaluation protocol. Table 4 compares different proposal generation methods in terms of grasp quality, hand–object penetration, and generation diversity.

As shown in Table 4a, GraspDiffusion achieves the best performance on both Seen $Q_{1}$ and Unseen $Q_{1}$, reaching 0.0464 ± 0.0011 and 0.0368 ± 0.0010, respectively. These results surpass those of the GraspGlow-based generative model used in UniDexGrasp, suggesting that the diffusion-based generation process can better capture the conditional distribution of grasp targets given the object point cloud, thereby producing higher-quality grasp candidates. For penetration, GraspDiffusion obtains the lowest value on seen-category objects, 0.187 ± 0.006, indicating stronger physical consistency between the generated grasp targets and object geometry. Although ProHMR+T achieves slightly lower penetration on unseen objects, its $Q_{1}$ and diversity scores are substantially worse than those of the proposed method. This suggests that minimizing penetration alone is insufficient to ensure the overall quality of generated grasps.

Table 4b further compares the grasp diversity of different methods. GraspTTA and DDG show limited rotational diversity, indicating that their predictions are concentrated around a small set of grasp patterns. Although ReLie + T and ProHMR + T achieve high diversity in certain rotation or keypoint metrics, their low $Q_{1}$ scores suggest that such diversity does not translate into high-quality grasps. In contrast, GraspDiffusion achieves the best or competitive results on $\sigma_{R}$, $\sigma_{T|R}$, $\sigma_{q|R}$, and $\sigma_{\mathrm{key}}$. This indicates that GraspDiffusion can generate high-quality grasp targets while covering a broader distribution of rotations, translations, and joint configurations.

**Figure 6:**
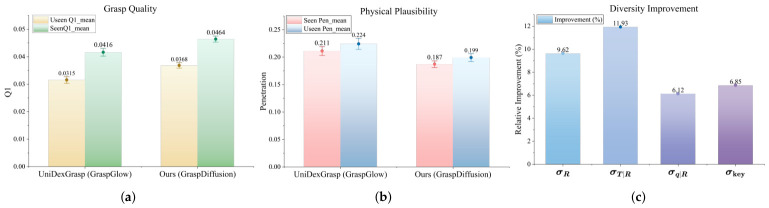
Comparison between GraspGlow and GraspDiffusion in stage-1 grasp proposal generation, where higher Q1 indicates better grasp proposal quality. (**a**) Grasp quality comparison on seen-category and unseen-category objects. (**b**) Physical plausibility comparison measured by hand–object penetration, where lower penetration indicates better physical feasibility. (**c**) Relative diversity improvement of GraspDiffusion over GraspGlow across rotation, translation-conditioned, joint-conditioned, and keypoint-level diversity metrics.

Figure 6 compares GraspDiffusion and GraspGlow in terms of grasp quality, physical feasibility, and diversity. As shown in Figure 6a, GraspDiffusion achieves higher Q1 scores on both seen and unseen object categories. In particular, the Q1 score on unseen categories increases from 0.0315 for GraspGlow to 0.0368. Since Q1 measures grasp contact quality and stability, this result suggests that the diffusion-based model can generate higher-quality grasp candidates conditioned on object point clouds, especially for objects outside the training categories.

Figure 6b shows that GraspDiffusion consistently reduces hand–object penetration. On unseen categories, penetration decreases from 0.224 to 0.199, suggesting better geometric consistency and improved physical feasibility. Figure 6c further evaluates the diversity of generated grasps. To enable a unified comparison across different diversity metrics, we report the relative improvement of GraspDiffusion over GraspGlow. For a diversity metric d, the relative improvement is defined as

(14) $$\mathrm{Improvement}\;\left(d\right)=\frac{d_{\mathrm{GraspDiffusion}}-d_{\mathrm{GraspGlow}}}{d_{\mathrm{GraspGlow}}+\epsilon }\times 100\%,$$

where $d_{\mathrm{GraspDiffusion}}$ and $d_{\mathrm{GraspGlow}}$ denote the metric values of GraspDiffusion and GraspGlow, respectively, and ϵ is a small constant introduced to avoid division by zero. Figure 6c shows that GraspDiffusion achieves consistent gains over GraspGlow on $\sigma_{R}$, $\sigma_{T|R}$, $\sigma_{q|R}$, and $\sigma_{\mathrm{key}}$ with relative improvements of 9.62%, 11.93%, 6.12%, and 6.85%, respectively. This indicates that diffusion-based grasp generation better captures the diversity of rotations, translations, joint angles, and hand keypoints. Combined with its performance in quality and physical feasibility, GraspDiffusion provides more reliable and diverse grasp targets for the target-conditioned execution policy.

### 4.4. Goal-Conditioned Policy Evaluation

This section evaluates the goal-conditioned execution capability of the Goal-Conditioned Diffusion Policy. Unlike end-to-end evaluations, whose performance is jointly affected by grasp proposal quality and execution policy, this experiment adopts an oracle-level setting to compare different execution strategies under given target grasp conditions. The goal is to examine whether each policy can generate stable actions and accurately complete the target grasp when the goal condition is provided. Therefore, this experiment specifically verifies the contribution of action-sequence diffusion modeling to the skillful grasp execution stage.

Table 5 summarizes the training paradigms and action representations of the compared execution strategies. The PPO [53] is trained with RL, the UniDexGrasp-S is trained with DAgger/behavior cloning, whereas our method uses diffusion-based behavior cloning and directly predicts an H-step action sequence. This comparison highlights the key difference between our method and single-step action policies.

As shown in Table 6, the proposed method achieves the highest success rate and the lowest MPE across all data partitions. On the Train, Test Seen-Cat, and Test Unseen-Cat splits, our method obtains success rates of 0.77 ± 0.015, 0.73 ± 0.018, and 0.69 ± 0.021, respectively, outperforming both the PPO Teacher and the UniDexGrasp Student. Notably, on unseen-category objects, our method improves the success rate from 0.59 ± 0.027 to 0.69 ± 0.021 compared with the UniDexGrasp Student, suggesting that diffusion-based action sequence modeling provides better generalization to novel object categories.

Our method also shows clear advantages in target execution accuracy. On the Test Unseen-Cat split, it achieves an MPE of 3.8 ± 0.25 cm, which is lower than that of the PPO Teacher 4.7 ± 0.32 cm and the UniDexGrasp Student 4.8 ± 0.32 cm. These results indicate that the target-conditional diffusion policy not only improves grasping success but also executes target grasps more accurately. Overall, this experiment supports the effectiveness of the second-stage policy design; compared with single-step action prediction, diffusion-based action sequence generation better models the temporal structure of dexterous grasping, leading to more stable and accurate target-conditioned execution.

### 4.5. Ablation Analysis

This section evaluates the contribution of key components in DexGraspDiffuser through ablation studies. Unlike the overall performance evaluation, these experiments focus on the effects of GraspDiffusion, target-conditioned inputs, diffusion policy, action sequence length, multi-candidate grasp sampling, and the receding-horizon execution mechanism on end-to-end grasp performance.

As shown in Table 7, the full model achieves the best overall performance, with success rates of 0.72 ± 0.019 and 0.68 ± 0.022 on Test Seen-Cat and Test Unseen-Cat, respectively. The corresponding MPE values are 3.6 ± 0.22 cm and 4.0 ± 0.25 cm, indicating accurate and robust grasp execution across both seen and unseen object categories. Removing GraspDiffusion reduces the success rate on unseen categories to 0.60 ± 0.027 and increases the MPE to 4.6 ± 0.33 cm, suggesting that high-quality target grasp generation in the first stage is important for reliable downstream execution.

The impact of the goal condition is particularly pronounced. Without the goal condition, the success rate on unseen categories drops further to 0.52 ± 0.035, while the MPE increases to 5.9 ± 0.46 cm, resulting in the largest performance degradation among all ablation settings. This result indicates that the target grasp is not merely an intermediate output of the first stage, but also a key conditioning signal that links geometric perception to action generation. Without explicit goal constraints, the policy becomes less capable of converging to stable and physically plausible grasp configurations.

The Diffusion Policy and temporal action modeling also make clear contributions. Removing the Diffusion Policy decreases the unseen-category success rate to 0.57 ± 0.030. Similarly, reducing the action horizon to a single-step prediction setting, H = 1, yields a success rate of 0.59 ± 0.029, both of which are notably lower than the full model. These results suggest that, compared with single-step action prediction, diffusion-based sequence generation better captures the continuous process of approaching the object, establishing contact, and closing the hand during dexterous grasping.

The ablations on S=1 and w/o RHC further show the complementary roles of multi-candidate grasp sampling and closed-loop replanning. When only one grasp target is sampled, the unseen-category success rate decreases to 0.63 ± 0.026, indicating that diverse grasp candidates improve target selection. Removing RHC reduces the success rate to 0.61 ± 0.029 and increases the MPE to 4.7 ± 0.35 cm, suggesting that receding-horizon execution helps correct actions under updated observations and contact disturbances. Overall, Table 7 confirms the necessity of each component. Among them, the goal condition produces the largest degradation when removed, while the Diffusion Policy and closed-loop execution are also critical for robust dexterous grasping.

### 4.6. Sensitivity Analysis of the RHC Smoothing Coefficient

To further examine the influence of the RHC smoothing coefficient, we conduct a sensitivity analysis by varying ρ among 0.05, 0.10, and 0.20. The smoothing coefficient controls the trade-off between action responsiveness and temporal smoothness in receding-horizon execution. A smaller ρ allows the policy to follow the newly predicted action more aggressively, but may introduce discontinuities between adjacent action horizons. In contrast, a larger ρ increases action smoothness, but may reduce the responsiveness of the policy to contact-induced deviations and updated observations.

As shown in Table 8, ρ=0.10 achieves the best overall performance across all three evaluation splits. When ρ=0.05, the policy remains more responsive to newly predicted actions, but the reduced smoothing leads to slightly lower success rates and higher MPE values, indicating less stable execution across adjacent receding-horizon windows. When ρ=0.20, stronger smoothing improves temporal inertia but weakens the ability to react to contact changes, resulting in larger performance degradation, especially on unseen-category objects. Therefore, ρ=0.10 provides the best trade-off between execution smoothness and closed-loop adaptability, and is used as the default setting in all main experiments.

### 4.7. Robustness Analysis Under Disturbances

This section evaluates the robustness of DexGraspDiffuser under perturbations, focusing on whether RHC can mitigate performance degradation caused by object pose changes, point cloud observation noise, and action execution bias. Since agile grasping involves continuous contact and coordinated multi-finger control, open-loop execution is prone to error accumulation. We therefore compare open-loop execution with RHC under different execution step sizes *M*.

As shown in Table 9, open-loop execution achieves low success rates under all three perturbation settings, with an average success rate of only 0.39 ± 0.034. In contrast, RHC consistently improves performance, suggesting that repeated observation and replanning during execution can help correct contact errors and state deviations. Among all settings, M=4 achieves the best overall performance, with success rates of 0.60 ± 0.024, 0.63 ± 0.026, and 0.58 ± 0.025 under object pose perturbation, point cloud noise, and action noise, respectively. Its average success rate reaches 0.60 ± 0.023.

The comparison across different execution step sizes shows that RHC requires a balance between motion continuity and feedback frequency. When M=8, the policy executes longer action segments before replanning, which delays feedback correction and reduces the average success rate to 0.54 ± 0.027. When M=2, feedback is more frequent, but overly frequent replanning can introduce discontinuities between consecutive action segments, leading to slightly lower performance than M=4. In addition, removing action smoothing decreases the average success rate from 0.60 ± 0.023 to 0.55 ± 0.030, indicating that smoothing helps reduce abrupt changes between adjacent replanning windows. Overall, these results show that RHC, together with motion smoothing, improves the closed-loop robustness of DexGraspDiffuser in perturbed environments.

## 5. Conclusions

This paper presented DexGraspDiffuser, a target-coupled two-stage conditional diffusion framework for goal-conditioned dexterous grasping. The framework first uses GraspDiffusion to generate point-cloud-conditioned grasp targets and then employs a Goal-Conditioned Diffusion Policy to produce temporally consistent action sequences for execution. During inference, receding-horizon execution enables action-prefix execution and online replanning, improving stability under contact-rich interactions. Simulation experiments demonstrate the effectiveness of the proposed framework. Across training objects, unseen objects from seen categories, and objects from unseen categories, DexGraspDiffuser achieves an average success rate of 0.72, with a train-to-unseen generalization gap of 0.08. Compared with the reproduced UniDexGrasp-T baseline under the same object split and evaluation protocol, DexGraspDiffuser improves the three-split average success rate by 3.3 percentage points and reduces the average mean position error by 0.53 cm. In grasp proposal evaluation, GraspDiffusion improves unseen-category $Q_{1}$ from 0.0315 to 0.0368 and reduces penetration from 0.224 to 0.199 compared with the GraspGlow-based baseline. It also improves diversity metrics by 6.12–11.93%. Oracle-level evaluation and ablation studies further confirm the contributions of goal conditioning, GraspDiffusion, action-sequence diffusion, multi-sample proposal generation, and receding-horizon execution. Under disturbance settings, RHC with M=4 improves the average success rate from 0.39 for open-loop execution to 0.60, indicating improved robustness to object perturbation, point cloud noise, and action noise.

Nevertheless, the proposed framework still has shortcomings and limitations that should be further addressed. In particular, this study has not yet included direct experimental comparisons with several very recent large-scale dexterous grasping systems, such as UniDexGrasp++, UniGraspTransformer, and AffordDex. These methods are highly relevant to the present work, but they are built upon different training assumptions and computational pipelines, including geometry-aware curriculum learning, large-scale policy distillation from object-specific reinforcement learning policies, and affordance-aware modeling with human priors. A rigorous comparison with these systems would require reproducing their complete training pipelines under matched datasets, simulator settings, trajectory budgets, and computational resources. Under our current experimental platform, such a comparison is difficult to conduct in a fully fair and reliable manner. Therefore, this paper focuses on controlled comparisons under a consistent goal-conditioned benchmark, where the proposed method seeks a practical balance among grasping success, expert-trajectory scale, and training cost.

In the future, we will address these limitations by actively extending our physical experimental platform to evaluate DexGraspDiffuser in real-world scenarios. Our team is currently establishing a dedicated robotic testing environment; however, due to hardware procurement cycles and platform construction time, full deployment remains a mid-to-long-term objective. Real-world deployment may be affected by several sim-to-real gap factors, including contact-dynamics mismatch, friction and compliance uncertainty, actuator calibration errors, and real point-cloud noise or occlusion patterns that are not fully captured by the simulated environment. To improve physical transferability, future work will incorporate domain randomization over object mass, friction, contact parameters, and sensor noise, together with tactile/proximity sensing fusion, system identification, and real-robot fine-tuning. Once completed, we intend to validate the proposed framework on physical dexterous hands in actual industrial pipelines. Beyond physical deployment, we will also investigate more challenging and practically relevant scenarios, including precise dexterous grasping in cluttered scenes, robust grasping under severe occlusion and contact uncertainty, and the integration of visual–tactile perception for fine-grained contact reasoning. In addition, incorporating vision-language models or embodied foundation models may further improve task-level grasp understanding and object affordance reasoning. With improved computational resources and a more scalable experimental setup, we aim to provide a broader evaluation of DexGraspDiffuser and further explore its potential for generalizable, bio-inspired dexterous manipulation.

## Figures and Tables

**Table 1:** Key experimental settings.

Item	Setting
GraspDiffusion steps	$K_{g}=1000$
Policy diffusion steps	$K_{a}=100$
Hidden dimension	256 for Stage 1, 512 for Stage 2
Beta schedule	Linear, $\beta_{1}=1\times 10^{-4},\beta_{k}=0.02$
Optimizer	Adam
Learning rate	$1\times 10^{-4}$
Weight decay	$1\times 10^{-4}$ for GraspDiffusion
Batch size	16 for GraspDiffusion, 256 for Diffusion Policy
Training epochs	100 for GraspDiffusion, 100 for Diffusion Policy
Grasp samples per object	S=8
Action horizon	H=16
Execution horizon	M=4
Smoothing coefficient	ρ=0.1
Number of cameras	5
Episode length	250
Control frequency	60 Hz
Random seeds	3

**Table 2:** End-to-end goal-conditioned dexterous grasping results.

Method	Train Succ ↑	Train MPE ↓ (cm)	Test Seen-Cat Succ ↑	Test Seen-Cat MPE ↓ (cm)	Test Unseen-Cat Succ ↑	Test Unseen-Cat MPE ↓ (cm)
MP	0.11±0.010	1.3±0.12	0.03±0.006	1.9±0.15	0.02±0.005	1.9±0.18
PPO [53]	0.15±0.017	4.5±0.28	0.12±0.014	5.0±0.35	0.10±0.012	5.7±0.42
DAPG [54]	0.14±0.016	7.8±0.47	0.12±0.017	7.6±0.45	0.10±0.014	8.9±0.58
ILAD [55]	0.24±0.019	5.2±0.31	0.21±0.021	5.4±0.33	0.19±0.018	5.7±0.39
UniDexGrasp-T [9]	0.73±0.019	3.6±0.21	0.69±0.023	4.1±0.26	0.64±0.027	4.7±0.31
UniDexGrasp-S [9]	0.66±0.022	4.0±0.25	0.62±0.024	4.5±0.29	0.58±0.026	4.9±0.34
**Ours**	0.76±0.016	3.2±0.18	0.72±0.019	3.6±0.22	0.68±0.022	4.0±0.25

**Table 3:** Description of grasp proposal evaluation metrics.

Metric	Description
$Q_{1}$	Grasp quality score for contact and stability
Pen.	Hand–object penetration score
$\sigma_{R}$	Diversity of hand root rotations
$\sigma_{T|R}$	Translation diversity conditioned on rotation
$\sigma_{q|R}$	Joint-angle diversity conditioned on rotation
$\sigma_{\mathrm{key}}$	Spatial diversity of hand keypoints

**Table 4:** Grasp proposal generation quality and diversity.

**(a) Grasp proposal quality.**				
**Method**	**Seen** $Q_{1}$ **↑**	**Seen Pen. ↓**	**Unseen** $Q_{1}$ **↑**	**Unseen Pen. ↓**
GraspTTA	0.0275±0.0012	0.360±0.014	0.0244±0.0011	0.368±0.016
DDG	0.0349±0.0016	0.326±0.011	0.0231±0.0013	0.342±0.014
ReLie + T	0.0196±0.0009	0.223±0.010	0.0194±0.0008	0.229±0.011
ProHMR + T	0.0214±0.0010	0.206±0.009	0.0226±0.0011	0.194±0.008
UniDexGrasp	0.0416±0.0014	0.211±0.008	0.0315±0.0012	0.224±0.010
**Ours**	0.0464±0.0011	0.187±0.006	0.0368±0.0010	0.199±0.007
**(b) Grasp proposal diversity.**				
**Method**	$\sigma_{R}$ **↑ (deg)**	$\sigma_{T|R}$ **↑ (cm)**	$\sigma_{q|R}$ **↑ (deg)**	$\sigma_{\mathrm{key}}$ **↑ (cm)**
GraspTTA	5.2±0.8	/	/	2.96±0.22
DDG	0.3±0.2	/	/	0.12±0.06
ReLie + T	108.4±3.8	/	/	6.52±0.31
ProHMR + T	86.9±4.2	/	/	5.73±0.28
UniDexGrasp	125.8±4.6	1.09±0.07	5.72±0.31	6.28±0.25
**Ours**	137.9±3.7	1.22±0.08	6.07±0.24	6.71±0.19

**Table 5:** Training paradigm and action representation of oracle-level execution policies.

Method	Training Paradigm	Action Form
PPO [53]	RL	Single-step action
UniDexGrasp-S [9]	DAgger/BC	Single-step action
**Ours**	BC diffusion	H-step action sequence

**Table 6:** Oracle-level goal-conditioned execution results.

Method	Train Succ ↑	Train MPE ↓ (cm)	Test Seen-Cat Succ ↑	Test Seen-Cat MPE ↓ (cm)	Test Unseen-Cat Succ ↑	Test Unseen-Cat MPE ↓ (cm)
PPO [53]	0.73±0.019	3.6±0.22	0.70±0.021	4.0±0.25	0.64±0.028	4.7±0.32
UniDexGrasp-S [9]	0.67±0.024	3.9±0.25	0.63±0.023	4.4±0.30	0.59±0.027	4.8±0.32
**Ours**	0.77±0.015	3.0±0.18	0.73±0.018	3.4±0.21	0.69±0.021	3.8±0.25

**Table 7:** Ablation study.

Variant	Test Seen-Cat Succ ↑	Test Seen-Cat MPE ↓ (cm)	Test Unseen-Cat Succ ↑	Test Unseen-Cat MPE ↓ (cm)
Full DexGrasp Diffuser	0.72±0.019	3.6±0.22	0.68±0.022	4.0±0.25
*w*/*o* GraspDiffusion	0.66±0.025	4.0±0.28	0.60±0.027	4.6±0.33
*w*/*o* Goal Condition	0.58±0.032	5.4±0.41	0.52±0.035	5.9±0.46
*w*/*o* Diffusion Policy	0.62±0.028	4.9±0.35	0.57±0.030	5.3±0.38
H=1 single-step policy	0.64±0.026	4.6±0.31	0.59±0.029	5.0±0.34
S=1 single grasp sample	0.68±0.022	3.9±0.26	0.63±0.026	4.3±0.30
*w*/*o* RHC	0.65±0.027	4.3±0.31	0.61±0.029	4.7±0.35

**Table 8:** Sensitivity analysis of the RHC smoothing coefficient ρ.

ρ	Train Succ ↑	Train MPE ↓ (cm)	Test Seen-Cat Succ ↑	Test Seen-Cat MPE ↓ (cm)	Test Unseen-Cat Succ ↑	Test Unseen-Cat MPE ↓ (cm)
0.05	0.73±0.023	3.42±0.21	0.69±0.022	3.86±0.31	0.65±0.019	4.28±0.30
0.10	0.76±0.016	3.20±0.18	0.72±0.019	3.60±0.22	0.68±0.022	4.00±0.25
0.20	0.70±0.028	3.56±0.23	0.68±0.024	4.02±0.29	0.64±0.017	4.43±0.32

**Table 9:** The robustness analysis under disturbances.

Variant	Object Perturb. Succ ↑	Point Cloud Noise Succ ↑	Action Noise Succ ↑	Average Succ ↑
Open-loop execution	0.37±0.038	0.42±0.033	0.39±0.036	0.39±0.034
RHC, M=8	0.53±0.030	0.56±0.031	0.52±0.028	0.54±0.027
RHC, M=4	0.60±0.024	0.63±0.026	0.58±0.025	0.60±0.023
RHC, M=2	0.58±0.031	0.61±0.029	0.57±0.030	0.59±0.029
RHC, M=4, w/o smoothing	0.55±0.028	0.58±0.033	0.53±0.031	0.55±0.030

## Data Availability

https://github.com/PKU-EPIC/UniDexGrasp/tree/main/dexgrasp_generation/datasets (accessed on 30 June 2026).
